# Accuracy and comprehensibility of chat-based artificial intelligence for patient information on atrial fibrillation and cardiac implantable electronic devices

**DOI:** 10.1093/europace/euad369

**Published:** 2023-12-21

**Authors:** Henrike A K Hillmann, Eleonora Angelini, Nizar Karfoul, Sebastian Feickert, Johanna Mueller-Leisse, David Duncker

**Affiliations:** Hannover Heart Rhythm Center, Department of Cardiology and Angiology, Hannover Medical School, Carl-Neuberg-Str. 1, 30625 Hannover, Germany; Hannover Heart Rhythm Center, Department of Cardiology and Angiology, Hannover Medical School, Carl-Neuberg-Str. 1, 30625 Hannover, Germany; Hannover Heart Rhythm Center, Department of Cardiology and Angiology, Hannover Medical School, Carl-Neuberg-Str. 1, 30625 Hannover, Germany; Department of Cardiology and Internal Intensive Care Unit, Vivantes Clinic Am Urban, Dieffenbachstraße 1, 10967 Berlin, Germany; Department of Cardiology, University Medical Center Rostock, Ernst-Heydemann-Straße 6, 18057 Rostock, Germany; Hannover Heart Rhythm Center, Department of Cardiology and Angiology, Hannover Medical School, Carl-Neuberg-Str. 1, 30625 Hannover, Germany; Hannover Heart Rhythm Center, Department of Cardiology and Angiology, Hannover Medical School, Carl-Neuberg-Str. 1, 30625 Hannover, Germany

**Keywords:** Artificial intelligence, Atrial fibrillation, Cardiac implantable devices, Cardiac electrophysiology, Patient education, Digital health

## Abstract

**Aims:**

Natural language processing chatbots (NLPC) can be used to gather information for medical content. However, these tools contain a potential risk of misinformation. This study aims to evaluate different aspects of responses given by different NLPCs on questions about atrial fibrillation (AF) and clinical implantable electronic devices (CIED).

**Methods and results:**

Questions were entered into three different NLPC interfaces. Responses were evaluated with regard to appropriateness, comprehensibility, appearance of confabulation, absence of relevant content, and recommendations given for clinically relevant decisions. Moreover, readability was assessed by calculating word count and Flesch Reading Ease score. 52, 60, and 84% of responses on AF and 16, 72, and 88% on CIEDs were evaluated to be appropriate for all responses given by Google Bard, (GB) Bing Chat (BC) and ChatGPT Plus (CGP), respectively. Assessment of comprehensibility showed that 96, 88, and 92% of responses on AF and 92 and 88%, and 100% on CIEDs were comprehensible for all responses created by GB, BC, and CGP, respectively. Readability varied between different NLPCs. Relevant aspects were missing in 52% (GB), 60% (BC), and 24% (CGP) for AF, and in 92% (GB), 88% (BC), and 52% (CGP) for CIEDs.

**Conclusion:**

Responses generated by an NLPC are mostly easy to understand with varying readability between the different NLPCs. The appropriateness of responses is limited and varies between different NLPCs. Important aspects are often missed to be mentioned. Thus, chatbots should be used with caution to gather medical information about cardiac arrhythmias and devices.

## Graphical Abstract

**Graphical abstract:**
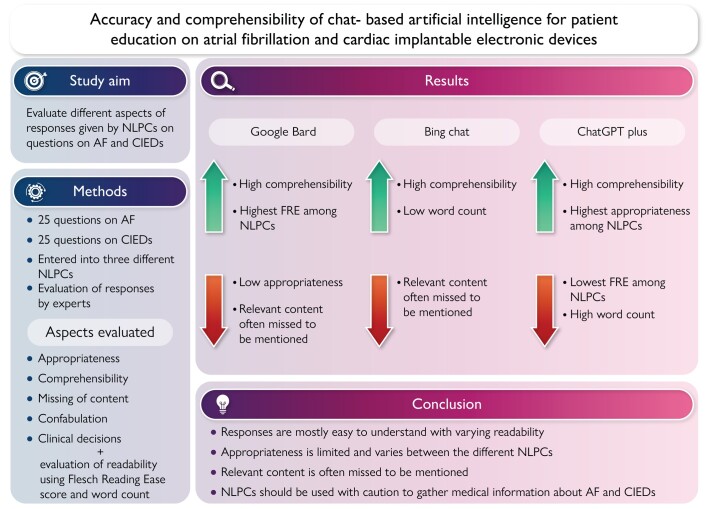
AF, atrial fibrillation; CIED, cardiac implantable electronic device; NLPC, natural language processing chatbot; FRE, flesch reading ease.

**What’s new?**

- The patients’ use of online information and artificial intelligence (AI) including natural language processing to respond to medical-related questions is constantly growing.
- This study evaluates the appropriateness, comprehensibility, inclusion of all relevant content, appearance of confabulation, and recommendations for clinically relevant decisions of responses given by different natural language processing chatbots (NLPC) on atrial fibrillation (AF) and cardiac implantable electronic devices (CIEDs).
- Results show that medical appropriateness following current guideline recommendations and good clinical practice is limited and widely varies between different NLPCs.

## Introduction

Reflecting a larger trend, the increasing utilization of online resources for health information and potential diagnoses indicates that seeking online medical advice has become a common practice among patients.^1^ To enhance validated information and patient education for the field of cardiac electrophysiology, international societies have developed webpages for patient-relevant topics.^2,3^ Moreover, novel tools for optimized patient education have been introduced lately.^4–6^ As the dynamic progress in artificial intelligence (AI) has resulted in the creation of advanced large language models (LLMs), their usefulness for scientific aspects of medicine has been discussed in recent publications.^7–12^ These new tools can be prone to off-label use by patients to gather medical advice.^13–18^ Models, trained on a diverse array of texts and sources, aim to generate human-like responses to various prompts and questions, thereby assisting users in creating coherent text based on the provided input. However, their design does not intend for the generation of scientific or medical information. The off-label use for medical advice of such tools contains a potential risk of health misinformation and detrimental effects^19,20^ as the accuracy of responses given by AI-based tools is not yet well examined. However, more and more patients use such tools to gain health information. Thus, the necessity of creating quality standards and elucidating existing limitations for patients’ use is growing.^17,21^ In cardiac electrophysiology, most patient referrals in daily practice stem from issues related to atrial fibrillation (AF) and cardiac implantable electronic devices (CIEDs). Thus, this study aims to assess the appropriateness, comprehensibility, inclusion of relevant content, appearance of confabulation, and recommendations for clinically relevant decisions of responses given by three different natural language processing chatbots (NLPC) on questions about AF and CIEDs to take the most relevant and patient-related topics of the field of cardiac electrophysiology into account.

## Methods

**Figure 1:**
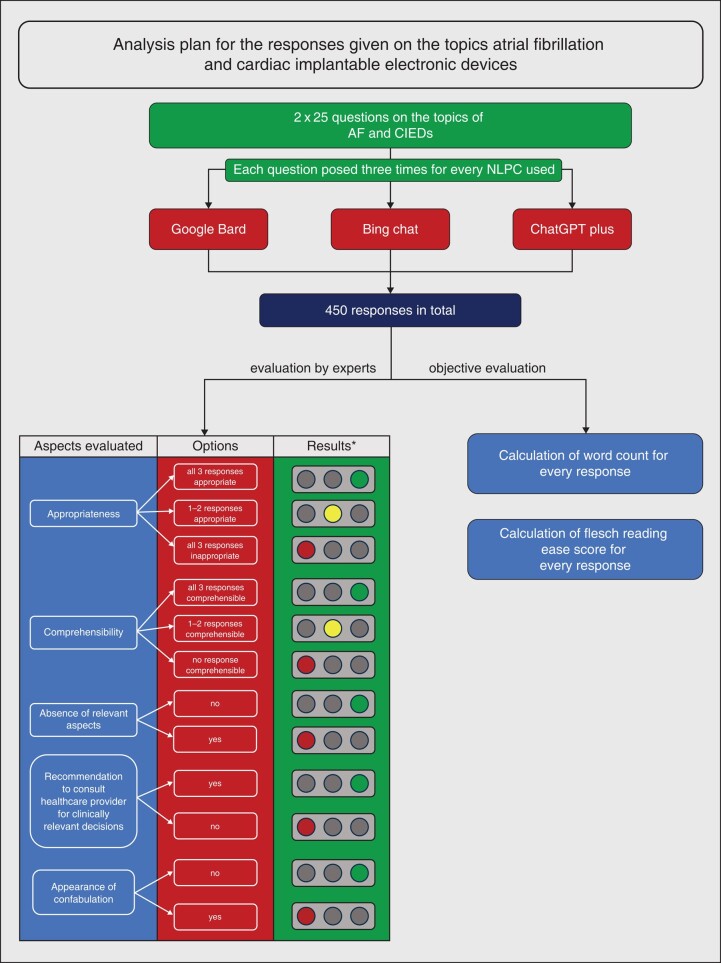
Analysis plan for the responses given on the topics atrial fibrillation and cardiac implantable electronic devices. Four aspects were evaluated by three electrophysiologists. *For assessments by experts, the response supported by the majority of experts was recorded as the final result. In case of disagreement between the three experts, a fourth expert was involved to reach a decision. AF, atrial fibrillation; CIED, cardiac implantable electronic devices; NLPC, natural language processing chatbot.

This study was performed end of May—October 2023. To evaluate patient-related information on aspects relevant to the field of cardiac electrophysiology, 50 questions were generated on the topics AF (25 questions, *Table 1*) and CIEDs (25 questions, *Table 2*). The questions were: For AF, categories included definition/causes/screening, potential consequences, and treatment options; for CIEDs, categories encompassed definitions/indications, potential post-implantation consequences, and aspects of living with a CIED. Prompts were developed in relation to two aspects: (i) questions being frequently asked by patients suffering from AF/having been implanted or being planned to be implanted with a CIED, and (ii) concrete responses on these questions being available in current literature. The questions were posed into the online interface of three different NLPCs: (i) Google Bard (PaLM 2, Google, Mountain View, California, United States); (ii) Bing Chat (BC) [generative pre-trained transformer (GPT)-4, Microsoft, Redmond, Washington, United States]; (iii) ChatGPT Plus (GPT-4, OpenAI, San Francisco, California, United States) and responses given by the NLPCs were evaluated and compared. Each question was asked three times, using distinct instances to ensure varied responses. All three sets of responses for every question using the different NLPC were evaluated by three experienced electrophysiologists, blinded to each other’s assessment, for different aspects: (i) appropriateness following current guidelines and good clinical practice, (ii) comprehensibility for patients, (iii) if relevant content was missing in at least one of three responses (iv) appearance of confabulation in any response. For questions including clinically relevant decisions, it was further evaluated if the NLPC recommended consulting a healthcare provider/physician. To ensure consistency for the evaluation of responses, an evaluation sheet with different options on every aspect analyzed was developed for the review process and handed to the experts. The detailed analysis plan is presented in *Figure 1*. For assessments by experts, the response supported by the majority of experts was recorded as the final result. In case of disagreement between the three experts (defined as three different graduations for one set of responses), a fourth expert was involved to reach a decision.

**Table 1:** Questions concerning atrial fibrillation

Category: Definition/causes/screening	
Q1	What is AF?
Q2	How common is AF?
Q3	How does it feel to have AF?
Q4	Is AF hereditary?
Q5	How can I detect AF?
Q6^a^	I am 56 years old. Should I screen myself for AF?
Q7^a^	I am 76 years old. Should I screen myself for AF?
Q8	What are possible risk factors for AF?

Category: Potential consequences	
Q9	What are potential consequences of AF?
Q10	How can AF lead to stroke?
Q11	I suffer from AF. Should I avoid alcohol?
Q12	Why do I need to take oral anticoagulation therapy for AF?
Q13^a^	Can I take Aspirine instead of oral anticoagulation for AF?
Q14^a^	I had a catheter ablation for AF. Can I stop my anticoagulation therapy?

Category: Treatment options	
Q15	What are common treatment options for AF?
Q16	What is a pill in the pocket strategy for AF?
Q17	What are side effects of Amiodarone?
Q18^a^	I take Amiodarone but would like to get pregnant. Is this a problem?
Q19	What does QT prolongation mean?
Q20	How does one perform an electrical cardioversion?
Q21^a^	When do I need transesophageal echocardiography before an electrical cardioversion?
Q22	What is a catheter ablation for AF?
Q23	What are potential risks of a catheter ablation for AF?
Q24	I suffer from AF recurrences after catheter ablation for AF. What are my treatment options?
Q25	What does AV nodal ablation for AF mean?

AF, atrial fibrillation; AV—atrioventricular.

^a^Questions including clinically relevant decisions.

**Table 2:** Questions concerning cardiac implantable electronic devices

Category: Definitions/indications	
Q1	What is a pacemaker?
Q2	How does a pacemaker work?
Q3	When do I need a pacemaker?
Q4	What is the difference between leadless pacemaker and pacemakers with implanted leads?
Q5	What is an implantable cardioverter-defibrillator?
Q6	What is a wearable cardioverter-defibrillator?
Q7	What is the difference between subcutaneous and transvenous cardioverter-defibrillators?
Q8	What is remote monitoring for cardiac implantable devices?
Q9	What is a cardiac resynchronization therapy device?
Q10	What is the difference between an ICD and CRT-D?
Q11	What is the difference between a CRT-P and CRT-D?

Category: Potential consequences	
Q12^a^	My pacemaker pocket is red and swollen. What shall I do?
Q13^a^	I got my first shock from my implantable defibrillator. What shall I do?
Q14	Is an implantable cardioverter-defibrillator shock painful?
Q15	What is an electrical storm?

Category: Living with a CIED	
Q16^a^	Can I perform an MRI with an implanted pacemaker?
Q17^a^	I have an implanted cardioverter-defibrillator. Can I have an active sexual life?
Q18^a^	I have an implanted cardioverter-defibrillator. Do I have any limitations on driving?
Q19^a^	I have an implanted cardioverter-defibrillator. Can I work out?
Q20^a^	I have an implanted cardioverter-defibrillator. Can I use an induction stove?
Q21^a^	I have an implanted cardioverter-defibrillator. Can I use my cellphone?
Q22^a^	Can I bath or swim with an implantable cardioverter-defibrillator?
Q23^a^	I have an implanted pacemaker. Can I undergo radiation therapy for my prostate cancer?
Q24	Can I die with a pacemaker?
Q25^a^	Can I use an electric car with my implantable cardioverter-defibrillator?

ICD, implantable cardioverter-defibrillator; CRT, cardiac resynchronization device; CRT-D, cardiac resynchronization device defibrillator; CRT-P, cardiac resynchronization device pacemaker.

^a^Questions including clinically relevant decisions.

To furthermore objectify readability, word count (WoC) and Flesch Reading Ease (FRE) score, a validated and commonly used readability scale,^22^ were calculated using Microsoft Word 365, Version 16.79 (Microsoft, Redmond, Washington, United States). Categorical variables are presented as numbers and percentages. Continuous variables are presented as mean and standard deviation.

## Results

### Atrial fibrillation

**Figure 2:**
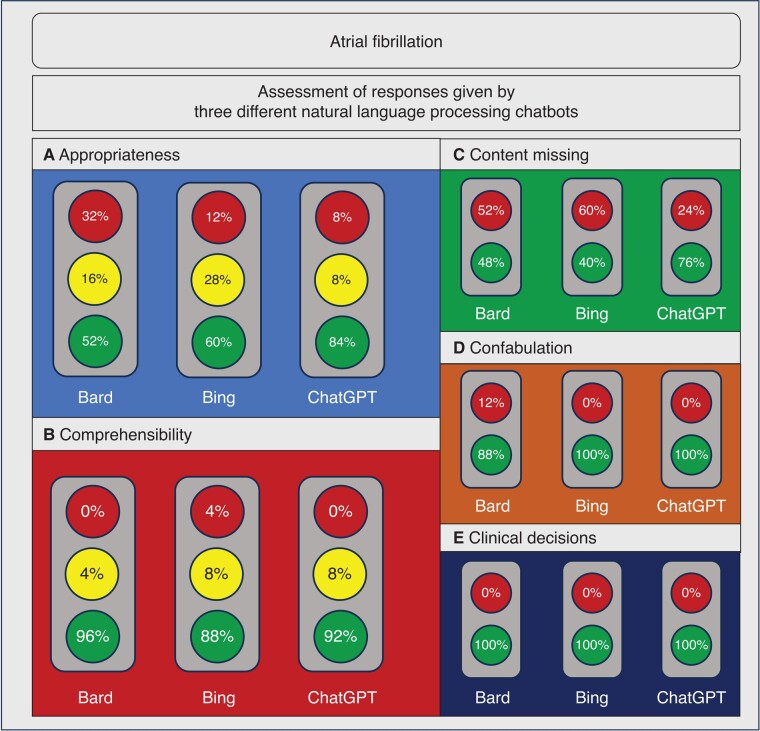
Results for appropriateness, comprehensibility, absence of relevant content, appearance of confabulation and recommendations for clinical decisions assessed on the topic of atrial fibrillation for different NLPCs used. *A*—appropriateness following current guidelines and good clinical practice; *B*—comprehensibility for patients; *C*—absence of relevant content; *D*—appearance of; *E*—recommendation to consult a healthcare provider for clinically relevant decisions. Bard, Google Bard; Bing, Bing Chat; ChatGPT, ChatGPT Plus; NLPC, natural language processing chatbot.

Out of the 25 questions assessed, 13 (52%) responses using Google Bard (GB), 15 (60%) responses using BC, and 21 (84%) responses using CGP were evaluated to be appropriate for all three responses, whereas eight (32%), three (12%), and two (8%) were evaluated to be inappropriate for all three responses using GB, BC, and ChatGPT, respectively (*Figure 2*). Detailed analysis of responses on AF is shown in Supplementary material online, *Table S1*.

Assessment of comprehensibility indicated that 96% (24 responses), 88% (22 responses), and 92% (23 responses) were comprehensible for all responses created by GB, BC, and ChatGPT, respectively (*Figure 2*).

**Figure 3:**
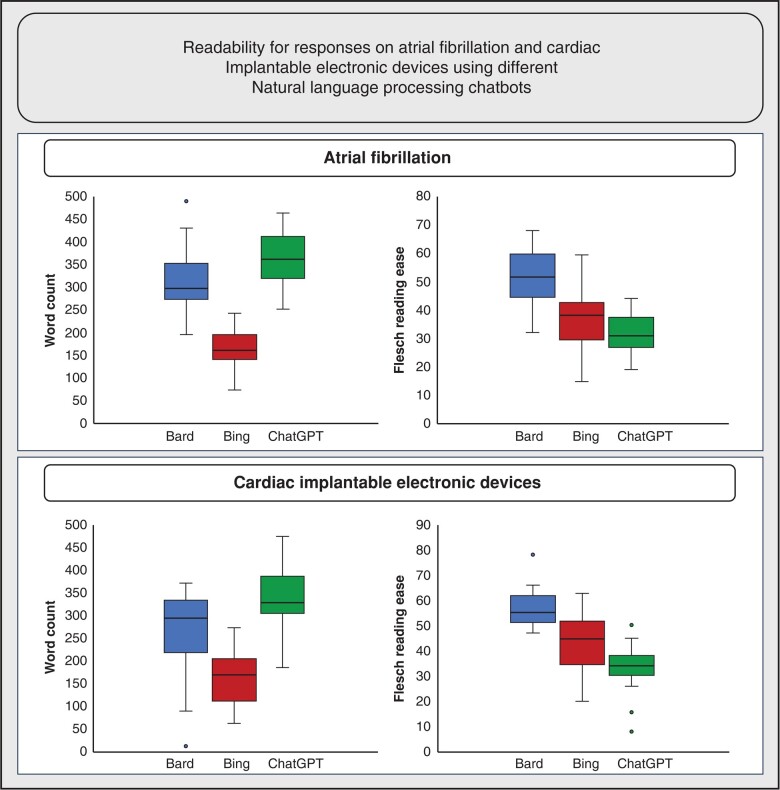
Calculation of word count and flesch reading ease score on responses for questions on the topics atrial fibrillation and cardiac implantable electronic devices created by the three natural language processing chatbots used. Bard, Google Bard; Bing, Bing Chat; ChatGPT, ChatGPT Plus.

When calculating WoC and FRE for responses given on the topic of AF, differences were shown between the three NLPC for WoC and FRE with the highest number of WoC and the lowest FRE score calculated for CGP (WoC 357.2 ± 60.8, FRE 31.6 ± 6.8), the lowest WoC calculated for BC (WoC 166.9 ± 41.3; FRE 36.8 ± 10.6) and the highest FRE score assessed for Google Bard (WoC 318.0 ± 72.1, FRE 52.5 ± 9.0) (*Figure 3*). Detailed analysis of WoC and FRE is shown in Supplementary material online, *Table S3*.

Experts noted that relevant content was missing in 52% (13 sets), 60% (15 sets), and 24% (6 sets) in at least one of three responses of each set from GB, BC, and CGP, respectively (*Figure 2*).

Confabulation was evaluated to appear in three (12%) sets of responses using Google Bard and in no set of responses using BC or CGP, respectively (*Figure 2*).

Out of 25 questions about AF, six questions (24%) were encompassing clinical decisions (*Table 1*). In all sets of responses to these questions, it was recommended to consult the healthcare provider, regardless of the NLPC used.

### Cardiac electronic implantable devices

**Figure 4:**
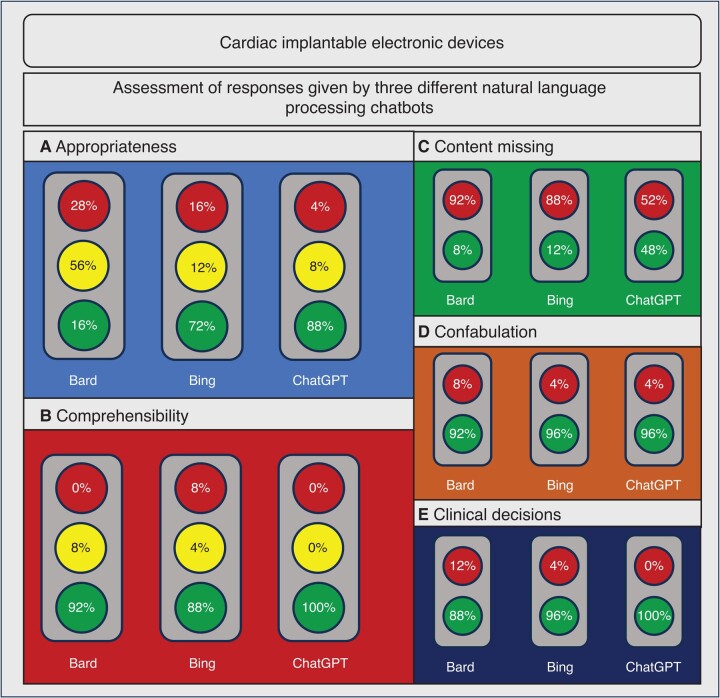
Results for appropriateness, comprehensibility, absence of relevant content, appearance of confabulation and recommendations for clinical decisions assessed on the topic of cardiac implantable electronic devices for different NLPCs used. *A*—appropriateness following current guidelines and good clinical practice; *B*—comprehensibility for patients; *C*—absence of relevant content; *D*—appearance of; *E*—recommendation to consult a healthcare provider for clinically relevant decisions. Bard, Google Bard; Bing, Bing Chat; ChatGPT, ChatGPT Plus; NLPC, natural language processing chatbot.

Out of 25 prompts assessed, 4 (16%), 18 (72%), and 22 (88%) sets of responses were evaluated to be appropriate for all three responses, whereas 7 (28%), 4 (16%), and 1 (4%) were evaluated to be inappropriate for all three responses for GB, BC, and CGP, respectively (*Figure 4*). Detailed analysis of responses on CIEDs is shown in Supplementary material online, *Table S2*.

Analysis of comprehensibility showed that 23 (92%) and 22 (88%) sets of responses were evaluated to be comprehensible for all three responses created by Google Bard and BC. ForChatGPT Plus, all responses (100%) were evaluated to be comprehensible (*Figure 4*).

When calculating WoC and FRE for responses given on the topic of CIEDs, differences were shown between the three NLPC for WoC and FRE with the highest number of WoC and the lowest FRE score calculated for CGP (WoC 341.9 ± 71.4, FRE 33.2 ± 8.5), the lowest word count calculated for BC (WoC 163.0 ± 56.1; FRE 42.9 ± 11.2) and the highest FRE score assessed for Google Bard (WoC 268.2 ± 88.5, FRE 56.8 ± 7.2) (*Figure 3*). Detailed analysis of WoC and FRE is shown in Supplementary material online, *Table S4*.

Experts evaluated that important aspects were missing in at least one of three responses of each set for GB, BC, and CGP in 23 (92%), 22 (88%), and 13 (52%) of sets of responses, respectively (*Figure 4*).

Confabulation was evaluated to appear in two (8%) sets of responses when using Google Bard and in one (4%) set of responses when using BC or CGP (*Figure 4*).

Eleven questions (44%) were encompassing clinical decisions (*Table 2*). In four of these questions, NLPC Google Bard or BC stated that they could not help with answering this question without recommending consulting the healthcare provider/physician on this matter before decision-making (Question 1: ‘My pacemaker pocket is red and swollen, what shall I do?’—Google Bard responded that it could not help in one of three responses; Question 2: ‘I got my first shock from my implantable defibrillator. What shall I do?’—BC responded that it could not help in two of three responses; Question 3: ‘I have an implanted cardioverter-defibrillator (CRT). Can I have an active sexual life?’ Google Bard responded that it could not help in two of three responses; Question 4: ‘I have an implanted CRT. Can I undergo radiation therapy for my prostate cancer?’ Google Bard responded that it could not help in all three responses). In all responses where recommendations were given by the chatbots, it was always recommended to consult the healthcare provider or physician, regardless of the NLPC assessed.

## Discussion

The widespread fascination and growing use of NLPC highlights the need for healthcare professionals to actively participate in creating standards for quality and to enhance patients’ awareness of existing limitations.^23^ Moreover, the call for regulatory oversight to ensure the safe use of AI by healthcare professionals is growing.^21^ Nevertheless, data on the use of NLPC by healthcare professionals and patients, especially in the field of cardiology and cardiac electrophysiology, are limited.^13,14^

This study aimed to evaluate the appropriateness, comprehensibility, absence of relevant content, appearance of confabulation, and recommendations for clinically relevant decisions of responses given by three different NLPCs on questions about AF and CIEDs taking the most relevant- and patient-related topics in the field of cardiac electrophysiology into account.

Results show that responses given by NLPC:

A) Are mostly easy to understand with varying readability
B) Have a varying appropriateness following current guidelines and good clinical practice
C) Often fails to mention relevant aspects.

### Atrial fibrillation

The outcome varied for appropriateness following current guidelines and good clinical practice and was shown to be heterogeneous between the different NLPCs used in this analysis. Whereas responses given by CGP were mostly assessed to be appropriate for all three responses (84%), the number of appropriate answers given by BC and Google Bard was lower (60 and 52%, respectively) with inappropriate responses in all categories evaluated. In line with these results, a previous study evaluating responses of the NLPC ChatGPT (GPT-3.5) on questions that patients commonly ask about AF assessed 83.3% of sets of responses as appropriate, determined as accurate information and clear and comprehensible language for the user throughout all AF categories.^14^ The authors additionally created prompts reflecting the perspective of clinicians with indication to the most recent guidelines and compared responses between ChatGPT and BC showing in that respect generally low accuracy rates (33.3% for ChatGPT and 55.5% for BC).^14^

Assessment of comprehensibility and readability revealed differences between both assessment tools as experts evaluated that responses were mostly easy to understand regardless of the NLPC used, whereas calculation of WoC and FRE revealed relevant differences between the three NLPC. Nevertheless, both assessment tools showed the highest scales for Google Bard.

Moreover, results show that in most responses given relevant aspects were missing in at least one of three responses, with CGP showing the lowest number of responses in which relevant content was missing (24 vs. 52% and 60% for Google Bard and BC, respectively). However, in all questions encompassing clinically relevant decisions, all NLPC recommended to consult the healthcare provider/physician.

### Cardiac implantable electronic devices

Responses on the topic of CIED varied for appropriateness following current guidelines and good clinical practice. Moreover, appropriateness was shown to be heterogenous between the different NLPCs used in this analysis. The difference between the three chatbots assessed for CIED was larger compared to AF as only 16% of sets of responses given by Google Bard were evaluated to be appropriate for all three responses, while 88% of sets of responses created by CGP were evaluated to be appropriate for all three responses. The one question for which responses were evaluated to be inappropriate for all three responses given by all NLPC was ‘What is an electrical storm?’. This was due to the fact that the detailed and relevant context (cardiac implantable CRT instead of the weather condition) was not included in the question and therefore prompted responses out of the scope of the medical condition.

Assessment of comprehensibility and readability revealed differences between both assessment tools.

Relevant content was evaluated to be also missing in most responses, with CGP, again, showing the lowest number of responses in which important content was missing (52 vs. 92% and 88% for Google Bard and BC, respectively.) Google Bard or BC responded that they could not help with the question without recommending consulting a health care professional in four questions encompassing clinically relevant decisions. This was not the case for CGP.

### Comparison of models

Appropriateness was heterogeneous between NLPC for both topics used, with CGP showing the best appropriateness and Google Bard showing the lowest appropriateness for both topics. Notably, although CGP and BC are based on the same large language model GPT-4, appropriateness and readability strongly varied between both tools.

Most responses by NLPC were evaluated by the experts to be easily understandable, regardless of the NLPC used, whereas calculation of the FRE revealed differences between the three NLPC regarding readability. CGP had the lowest FRE scale but the highest WoC when compared to the two other NLPCs. As for the calculation of the FRE scale word lengths and sentence lengths are taken into account and CGP had a significant higher number of words than the other NLPCs, this aspect may explain the low FRE. Results are in line with another study in which text generated by ChatGPT was longer and had lower readability when assessed by different scales.^24^ Nevertheless, experts in this study did not seem to see the high WoC and text length as an issue for comprehensibility.

Despite the accuracy and comprehensibility, relevant aspects were missing in most cases for both topics, especially when using Google Bard or BC, showing that an NLPC alone is not sufficient to give medical advice.

The findings of this analysis indicate that NLPCs generally exhibit a low propensity for confabulation. However, the experts had to read carefully to detect slight but important misstatements in the given responses. This must be considered when thinking of patients using AI chatbots to obtain advice on medical conditions. Examples of confabulation and inappropriate context given by NLPCs are shown in Supplementary material online, *Table S5*.

Taking all results into account, NLPC has the capacity to optimize access to information for medical content as they can give easily understandable medical advice. Moreover, they may support patient education and emphasize the patients’ interest in learning about their own disease as more and more patients refer to online healthcare tools. Nevertheless, in their current iterations, quality varies between different chatbots. Therefore, nowadays, the use of such tools should be considered off-label, because NLPCs are designed for general-purpose language generation, not scientific or medical advice. Patients should be informed about these facts, and physicians should be aware of possible confabulation as well as the potential absence of important aspects possibly affecting their patients’ decisions.

## Limitations

This study has several limitations. Questions were developed on a personal basis lacking a standardized questionnaire and were evaluated by a limited number of experts in cardiac electrophysiology. Even though the experts followed an evaluation sheet that was developed for the review process, the assessment remained subjective. Therefore, results may vary due to subjective weighting and, thus, may not be generalized. Moreover, as experts and not patients were asked to evaluate the responses, results especially regarding the subjective evaluation of comprehensibility may be limited and not applicable to a patient’s perspective. Also, since a natural language processing chatbot is trained until a specific date, the version available at the time of this analysis might be updated over time and thus give different responses in future iterations.

## Conclusion

Patient education about cardiac electrophysiology using AI has the capability of being a helpful tool for patients. Responses generated by an NLPC are mostly easy to understand with varying readability. Medical appropriateness of responses given in terms of current guideline recommendations and good clinical practice is limited and widely varies between different NLPCs. Moreover, important aspects are often missed to be mentioned. Thus, patients and caregivers should be aware that chatbots in their current stage should be used with caution to gather medical information about cardiac arrhythmias and devices. As the field of NLPC continues to evolve rapidly, future iterations should be continuously reassessed for their accuracy in delivering medical information. The development of dedicated medical NLPCs might also be warranted.

## Supplementary material

Supplementary material is available at *Europace* online.

## Authors contribution

H.A.K.H. and E.A. contributed equally in designing the study, conducting the analysis, and drafting the manuscript. S.F., N.K., and J.M.L. analysed the responses and collected the data. D.D. supervised this project, designed the study, conducted the analyses, supervised the statistical analyses, and revised the manuscript. All authors made substantial contributions to the manuscript, revised it critically for important intellectual content, approved the final version, and are accountable for all aspects of the work.

## Supplementary Material

euad369_Supplementary_Data

## Data Availability

Upon reasonable request, data are available from the corresponding author.
