# Supplementary material for: Accuracy and comprehensibility of chat-based artificial intelligence for patient information on atrial fibrillation and cardiac implantable electronic devices
Source: Europace. 2023 Dec 21;26(1):euad369. doi: 10.1093/europace/euad369 (PMC10824484; doi:10.1093/europace/euad369)
Supplement: euad369_Supplementary_Data [file euad369_supplementary_data.docx]

Supplementary material

Accuracy and comprehensibility of chat-based artificial intelligence for patient information on atrial fibrillation and cardiac implantable electronic devices

Henrike A. K. Hillmann, MD^1+^; Eleonora Angelini, MD^1+^; Nizar Karfoul, MD^1^; Sebastian Feickert, MD^2^; Johanna Mueller-Leisse, MD, MSc^1^; David Duncker, MD^1*^

Affiliations:

^1^ Hannover Heart Rhythm Center, Department of Cardiology and Angiology, Hannover Medical School, Hannover, Germany

^2^ Department of Cardiology, Vivantes Klinikum Am Urban, Berlin and Rostock University Medical Center, Rostock, Germany

+ Both authors contributed equally to the manuscript and share first authorship.

Supplementary Table S1: Assessment of appropriateness, comprehensibility, absence of relevant content and appearance of confabulation for responses given on the topic of atrial fibrillation.

|  | Appropriate-ness | | | Comprehensibi-lity | | | Content missing | | | | Confabula-tion | | |
| --- | --- | --- | --- | --- | --- | --- | --- | --- | --- | --- | --- | --- | --- |
|  | Bard | Bing | ChatGPT | Bard | Bing | ChatGPT | Bard | Bing | | ChatGPT | Bard | Bing | ChatGPT |
| Definition/causes/screening |  | | |  | | |  | | | |  | | |
| What is AF? | 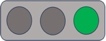 | 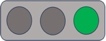 | 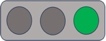 | 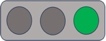 | 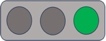 | 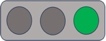 | Y | | Y | N | N | N | N |
| How common is AF?* | 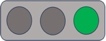 | 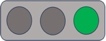 | 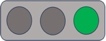 | 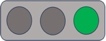 | 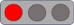 | 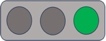 | N | N | | N | N | N | N |
| How does it feel to have AF? | 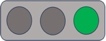 | 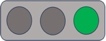 | 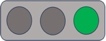 | 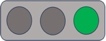 | 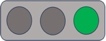 | 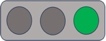 | N | N | | N | N | N | N |
| Is AF hereditary? | 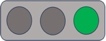 | 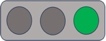 | 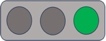 | 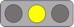 | 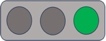 | 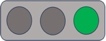 | N | N | | N | N | N | N |
| How can I detect AF? | 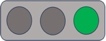 | 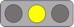 | 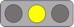 | 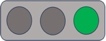 | 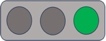 | 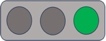 | Y | Y | | Y | N | N | N |
| I am 56 years old. Should I screen myself for AF? | 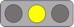 | 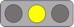 | 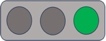 | 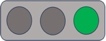 | 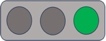 | 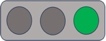 | Y | Y | | Y | N | N | N |
| I am 76 years old. Should I screen myself for AF? | 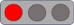 | 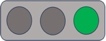 | 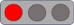 | 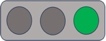 | 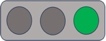 | 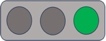 | Y | N | | Y | N | N | N |
| What are possible risk factors for AF? | 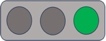 | 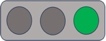 | 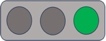 | 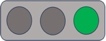 | 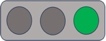 | 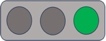 | N | Y | | N | N | N | N |
| Potential consequences |  | | |  | | |  | | | |  | | |
| What are potential consequences of AF?* | 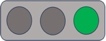 | 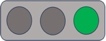 | 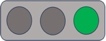 | 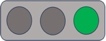 | 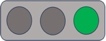 | 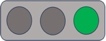 | N | N | | N | N | N | N |
| How can AF lead to stroke? | 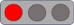 | 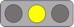 | 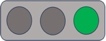 | 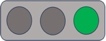 | 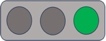 | 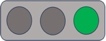 | Y | Y | | N | Y | N | N |
| I suffer from AF. Should I avoid alcohol?* | 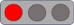 | 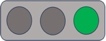 | 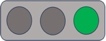 | 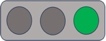 | 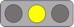 | 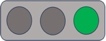 | N | N | | N | Y | N | N |
| Why do I need to take oral anticoagulation therapy for AF? | 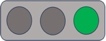 | 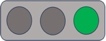 | 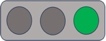 | 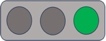 | 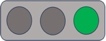 | 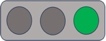 | N | N | | N | N | N | N |
| Can I take Aspirine instead of oral anticoagulation for AF? | 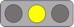 | 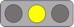 | 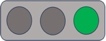 | 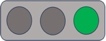 | 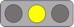 | 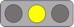 | N | N | | N | Y | N | N |
| I had a catheter ablation for AF. Can I stop my anticoagulation therapy? | 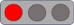 | 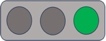 | 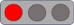 | 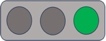 | 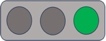 | 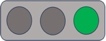 | N | N | | Y | N | N | N |
| Treatment options |  | | |  | | |  | | | |  | | |
| What are common treatment options for AF?* | 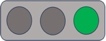 | 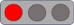 | 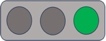 | 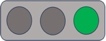 | 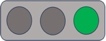 | 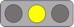 | N | Y | | Y | N | N | N |
| What is a pill in the pocket strategy for AF? | 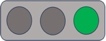 | 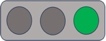 | 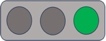 | 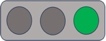 | 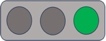 | 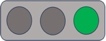 | Y | Y | | Y | N | N | N |
| What are side effects of Amiodarone?* | 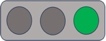 | 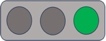 | 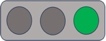 | 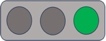 |  |  | Y | Y | | N | N | N | N |
| I take Amiodarone but would like to get pregnant. Is this a problem? * |  |  |  |  |  |  | Y | Y | | N | N | N | N |
| What does QT prolongation mean? |  |  |  |  |  |  | Y | Y | | N | N | N | N |
| How does one perform an electrical cardioversion?* |  |  |  |  |  |  | Y | Y | | N | N | N | N |
| When do I need transesophageal echocardiography before an electrical cardioversion? * |  |  |  |  |  |  | N | Y | | N | N | N | N |
| What is a catheter ablation for AF? |  |  |  |  |  |  | Y | Y | | N | N | N | N |
| What are potential risks of a catheter ablation for AF? |  |  |  |  |  |  | Y | Y | | N | N | N | N |
| I suffer from AF recurrences after catheter ablation for AF. What are my treatment options? |  |  |  |  |  |  | Y | Y | | N | N | N | N |
| What does AV nodal ablation for AF mean? |  |  |  |  |  |  | N | N | | N | N | N | N |

AF – atrial fibrillation, AV – atrioventricular, Y – Yes, N - No. * - questions in which a fourth expert was involved.

Supplementary Table S2: Assessment of appropriateness, comprehensibility, missing content, and appearance of confabulation for responds given on the topic of cardiac electronic implantable devices.

|  | Appropriate-ness | | | Comprehensibility | | | Content missing | | | Confabu-  lation | | |
| --- | --- | --- | --- | --- | --- | --- | --- | --- | --- | --- | --- | --- |
|  | Bard | Bing | ChatGPT | Bard | Bing | ChatGPT | Bard | Bing | ChatGPT | Bard | Bing | ChatGPT |
| Definitions/indications |  | | |  | | |  | | |  | | |
| What is a pacemaker? |  |  |  |  |  |  | Y | Y | Y | N | N | N |
| How does a pacemaker work? |  |  |  |  |  |  | Y | N | Y | N | N | N |
| When do I need a pacemaker? |  |  |  |  |  |  | N | N | N | N | N | N |
| What is the difference between leadless pacemaker and pacemakers with implanted leads?* |  |  |  |  |  |  | Y | Y | N | N | N | N |
| What is an implantable cardioverter-defibrillator?* |  |  |  |  |  |  | Y | Y | Y | N | N | N |
| What is a wearable cardioverter-defibrillator? |  |  |  |  |  |  | Y | Y | N | N | N | N |
| What is the difference between subcutaneous and transvenous cardioverter-defibrillators? |  |  |  |  |  |  | Y | N | N | N | N | N |
| What is remote monitoring for cardiac implantable devices? |  |  |  |  |  |  | Y | Y | N | N | N | N |
| What is a cardiac resynchronization therapy device?* |  |  |  |  |  |  | Y | Y | Y | N | N | N |
| What is the difference between an ICD and CRT-D? |  |  |  |  |  |  | Y | Y | N | N | N | N |
| What is the difference between an CRT-P and CRT-D?* |  |  |  |  |  |  | Y | Y | Y | N | N | N |
| Potential consequences |  | | |  | | |  | | |  | | |
| My pacemaker pocket is red and swollen. What shall I do? |  |  |  |  |  |  | Y | Y | N | N | N | N |
| I got my first shock from my implantable defibrillator. What shall I do? |  |  |  |  |  |  | Y | Y | Y | N | Y | N |
| Is an implantable cardioverter-defibrillator shock painful? |  |  |  |  |  |  | Y | Y | N | Y | N | N |
| What is an electrical storm? |  |  |  |  |  |  | Y | Y | Y | N | N | N |
| Living with an CIED |  | | |  | | |  | | |  | | |
| Can I perform an MRI with an implanted pacemaker? |  |  |  |  |  |  | Y | Y | Y | N | N | N |
| I have an implanted cardioverter-defibrillator. Can I have an active sexual life? |  |  |  |  |  |  | Y | Y | N | N | N | N |
| I have an implanted cardioverter-defibrillator. Do I have any limitations on driving?* |  |  |  |  |  |  | Y | Y | Y | N | N | N |
| I have an implanted cardioverter-defibrillator. Can I work out?* |  |  |  |  |  |  | Y | Y | Y | N | N | N |
| I have been implanted with a cardioverter-defibrillator. Can I use an induction stove? |  |  |  |  |  |  | Y | Y | Y | N | N | N |
| I have an implanted cardioverter-defibrillator. Can I use my cellphone? |  |  |  |  |  |  | Y | Y | N | N | N | N |
| Can I bath or swim with an implantable cardioverter-defibrillator? |  |  |  |  |  |  | Y | Y | Y | Y | N | Y |
| I have an implanted pacemaker. Can I undergo radiation therapy for my prostate cancer? |  |  |  |  |  |  | Y | Y | N | N | N | N |
| Can I die with a pacemaker? |  |  |  |  |  |  | Y | Y | N | N | N | N |
| Can I use an electric car with my implantable cardioverter-defibrillator? |  |  |  |  |  |  | N | Y | Y | N | N | N |

ICD – implantable cardioverter defibrillator, CRT – cardiac resynchronization therapy, CRT-D – cardiac resynchronization therapy with defibrillator, CRT-P – cardiac resynchronization therapy with pacemaker, Y – Yes, N - No. * - questions in which a fourth expert was involved.

Supplementary Table S3: Calculation of word count and Flesch Reading Ease score for responses given on the topic of atrial fibrillation.

|  | Language Model | Word count  (mean ± SD) | Flesch Reading Ease score (mean ± SD) |
| --- | --- | --- | --- |
| Definition/causes/screening |  |  |  |
| What is AF? | Bard | 291 ± 71.6 | 60.8 ± 4.2 |
|  | Bing | 161 ± 20.2 | 40.1 ± 4.4 |
|  | ChatGPT | 427 ± 28.5 | 35.8 ± 8.1 |
| How common is AF? | Bard | 196 ± 23.6 | 45.3 ± 3.3 |
|  | Bing | 152 ± 8.1 | 42 ± 0.8 |
|  | ChatGPT | 254 ± 25.1 | 30.2 ± 2 |
| How does it feel to have AF? | Bard | 287 ± 18.9 | 66.3 ± 2.6 |
|  | Bing | 149 ± 60.1 | 41.5 ± 2.4 |
|  | ChatGPT | 308 ± 28.9 | 44.1 ± 2.7 |
| How can I detect AF? | Bard | 431 ± 83.6 | 62.2 ± 5.3 |
|  | Bing | 222 ± 42.1 | 43.1 ± 3.6 |
|  | ChatGPT | 423 ± 41.0 | 42.8 ± 3.1 |
| Is AF hereditary? | Bard | 221 ± 21.9 | 51.6 ± 5.3 |
|  | Bing | 143 ± 0.6 | 35.9 ± 2.8 |
|  | ChatGPT | 252 ± 62.0 | 23.8 ± 3.9 |
| I am 56 years old. Should I screen myself for AF? | Bard | 428 ± 71.1 | 58.9 ± 5.9 |
|  | Bing | 139 ± 31.2 | 36.4 ± 2.4 |
|  | ChatGPT | 338 ± 74.2 | 37.4 ± 6.1 |
| I am 76 years old. Should I screen myself for AF? | Bard | 411 ± 27.5 | 61.3 ± 5.7 |
|  | Bing | 74 ± 18.4 | 24.3 ± 1.2 |
|  | ChatGPT | 321 ± 20.7 | 38.3 ± 4.9 |
| What are possible risk factors for AF? | Bard | 279 ± 22.9 | 52 ± 3.6 |
|  | Bing | 195 ± 14.2 | 46.9 ± 5.3 |
|  | ChatGPT | 362 ± 27.4 | 32.1 ± 2.2 |
| Potential consequences |  |  |  |
| What are potential consequences of AF? | Bard | 310 ± 14.5 | 56.8 ± 2.9 |
|  | Bing | 197 ± 11.2 | 59.5 ± 1.8 |
|  | ChatGPT | 373 ± 17.0 | 39.2 ± 2.5 |
| How can AF lead to stroke? | Bard | 346 ± 61.7 | 68.1 ± 2.1 |
|  | Bing | 203 ± 48.0 | 51 ± 7.2 |
|  | ChatGPT | 335 ± 28.0 | 37.5 ± 6.3 |
| I suffer from AF. Should I avoid alcohol? | Bard | 281 ± 50.0 | 60.6 ± 3.9 |
|  | Bing | 157 ± 17.3 | 38.2 ± 10.7 |
|  | ChatGPT | 290 ± 18.6 | 34.1 ± 2.6 |
| Why do I need to take oral anticoagulation therapy for AF? | Bard | 298 ± 26.8 | 58.6 ± 3.4 |
|  | Bing | 152 ± 47.4 | 28.9 ± 4.1 |
|  | ChatGPT | 402 ± 30.9 | 31 ± 3.5 |
| Can I take aspirin instead of oral anticoagulation for AF? | Bard | 244 ± 14.2 | 59 ± 7.7 |
|  | Bing | 123 ± 20.4 | 14.8 ± 5.4 |
|  | ChatGPT | 326 ± 64.0 | 28.3 ± 5.7 |
| I had a catheter ablation for AF. Can I stop my anticoagulation therapy? | Bard | 320 ± 24.6 | 32.1 ± 9.1 |
|  | Bing | 178 ± 22.9 | 22.8 ± 4.5 |
|  | ChatGPT | 322 ± 15.4 | 29.2 ± 2.7 |
| Treatment options |  |  |  |
| What are common treatment options for AF? | Bard | 490 ± 229.2 | 51.7 ± 3.3 |
|  | Bing | 225 ± 9.2 | 36.7 ± 1.1 |
|  | ChatGPT | 445 ± 29.6 | 29.5 ± 9.6 |
| What is a pill in the pocket strategy for AF? | Bard | 354 ± 33.0 | 55.4 ± 8.6 |
|  | Bing | 173 ± 5.5 | 15.2 ± 1.1 |
|  | ChatGPT | 367 ± 9.5 | 30.4 ± 4.2 |
| What are side effects of Amiodarone? | Bard | 261 ± 21.7 | 42.9 ± 9.9 |
|  | Bing | 128 ± 9.1 | 42.3 ± 3.4 |
|  | ChatGPT | 319 ± 27 | 20.9 ± 3.7 |
| I take Amiodarone but would like to get pregnant. Is this a problem? | Bard | 292 ± 34.8 | 41.2 ± 3.1 |
|  | Bing | 148 ± 62.7 | 30.2 ± 1 |
|  | ChatGPT | 266 ± 25.5 | 22.3 ± 7.4 |
| What does QT prolongation mean? | Bard | 291 ± 56.5 | 43.7 ± 9.4 |
|  | Bing | 181 ± 31.4 | 39.9 ± 3.8 |
|  | ChatGPT | 325 ± 62.0 | 23.5 ± 7 |
| How does one perform an electrical cardioversion? | Bard | 268 ± 79.4 | 50.7 ± 7.4 |
|  | Bing | 243 ± 14.0 | 46.6 ± 1.8 |
|  | ChatGPT | 464 ± 32.6 | 39.7 ± 3.7 |
| When do I need transesophageal echocardiography before an electrical cardioversion? | Bard | 301 ± 69.0 | 51.6 ± 5.7 |
|  | Bing | 96 ± 23.7 | 26.8 ± 2.1 |
|  | ChatGPT | 362 ± 28.3 | 19.1 ± 1.7 |
| What is a catheter ablation for AF? | Bard | 426 ± 82.0 | 50.1 ± 3.3 |
|  | Bing | 233 ± 26.1 | 41.9 ± 5.2 |
|  | ChatGPT | 381 ± 20.2 | 32.1 ± 2.6 |
| What are potential risks of a catheter ablation for AF? | Bard | 250 ± 64.5 | 38.8 ± 11.2 |
|  | Bing | 187 ± 9.5 | 34.9 ± 9.9 |
|  | ChatGPT | 436 ± 28.9 | 29.9 ± 3.4 |
| I suffer from AF recurrences after catheter ablation for AF. What are my treatment options? | Bard | 352 ± 21.6 | 43.8 ± 6.6 |
|  | Bing | 138 ± 9.2 | 34.3 ± 1.1 |
|  | ChatGPT | 434 ± 7.6 | 25.5 ± 2.8 |
| What does AV nodal ablation for AF mean? | Bard | 321 ± 13.9 | 48.6 ± 5.6 |
|  | Bing | 176 ± 24.6 | 45.9 ± 5.4 |
|  | ChatGPT | 397 ± 13.4 | 33.6 ± 3.8 |

AF – Atrial fibrillation; AV – atrioventricular; SD – standard deviation.

Supplementary Table S4: Calculation of word count and Flesch Reading Ease score for responses given on the topic of cardiac implantable electronic devices.

|  | Language Model | Word count  (mean ± SD) | Flesch Reading Ease score (mean ± SD) |
| --- | --- | --- | --- |
| Definitions/indications |  |  |  |
| What is a pacemaker? | Bard | 215 ± 44.5 | 49.3 ± 0.5 |
|  | Bing | 218 ± 2.9 | 55.1 ± 0.9 |
|  | ChatGPT | 390 ± 81.8 | 38 ± 3 |
| How does a pacemaker work? | Bard | 331 ± 6.6 | 47.2 ± 2.2 |
|  | Bing | 274 ± 40.7 | 49.5 ± 3.9 |
|  | ChatGPT | 434 ± 47.4 | 38.5 ± 2.5 |
| When do I need a pacemaker? | Bard | 295 ± 56.6 | 49.9 ± 7.3 |
|  | Bing | 206 ± 69.2 | 52.8 ± 6.8 |
|  | ChatGPT | 385 ± 24.8 | 35.3 ± 5.2 |
| What is the difference between leadless pacemaker and pacemakers with implanted leads? | Bard | 372 ± 24.1 | 51.2 ± 6.4 |
|  | Bing | 209 ± 40.2 | 38.9 ± 10.6 |
|  | ChatGPT | 469 ± 40.1 | 32.1 ± 1.7 |
| What is an implantable cardioverter-defibrillator? | Bard | 299 ± 75.5 | 55.8 ± 2.9 |
|  | Bing | 127 ± 57.7 | 20.1 ± 6.9 |
|  | ChatGPT | 380 ± 53.9 | 30.1 ± 3.2 |
| What is a wearable cardioverter-defibrillator? | Bard | 294 ± 7.2 | 52.4 ± 2.3 |
|  | Bing | 208 ± 28.7 | 43.3 ± 3.1 |
|  | ChatGPT | 377 ± 46.6 | 34.3 ± 7.9 |
| What is the difference between subcutaneous and transvenous cardioverter-defibrillators? | Bard | 245 ± 30.0 | 50.8 ± 7.5 |
|  | Bing | 170 ± 25.7 | 20.3 ± 6.1 |
|  | ChatGPT | 475 ± 15.0 | 34.2 ± 2.3 |
| What is remote monitoring for cardiac implantable devices? | Bard | 332 ± 97.4 | 50.5 ± 2.4 |
|  | Bing | 172 ± 23.3 | 33.9 ± 6.6 |
|  | ChatGPT | 399 ± 55.1 | 15.8 ± 0.2 |
| What is a cardiac resynchronization therapy device? | Bard | 357 ± 28.9 | 53.8 ± 2 |
|  | Bing | 243 ± 29.5 | 37.3 ± 2.8 |
|  | ChatGPT | 356 ± 18.6 | 35.9 ± 1.2 |
| What is the difference between an ICD and CRT-D? | Bard | 336 ± 44.5 | 59.8 ± 4 |
|  | Bing | 193 ± 15.9 | 62.9 ± 3 |
|  | ChatGPT | 186 ± 54.8 | 34 ± 7.6 |
| What is the difference between an CRT-P and CRT-D? | Bard | 299 ± 20.6 | 55.9 ± 5.8 |
|  | Bing | 202 ± 7.5 | 47 ± 1.4 |
|  | ChatGPT | 326 ± 78.0 | 31.4 ± 1.6 |
| Potential consequences |  |  |  |
| My pacemaker pocket is red and swollen. What shall I do? | Bard | 182 ± 145.2 | 51.9 ± 6.5 |
|  | Bing | 63 ± 6.9 | 53.3 ± 0.3 |
|  | ChatGPT | 200 ± 56.0 | 32.6 ± 4 |
| I got my first shock from my implantable defibrillator. What shall I do? | Bard | 354 ± 19.1 | 62.1 ± 3.7 |
|  | Bing | 103 ± 100.5 | 57.6 ± 10.4 |
|  | ChatGPT | 343 ± 46.2 | 50.4 ± 0.3 |
| Is an implantable cardioverter-defibrillator shock painful? | Bard | 224 ± 44.4 | 61.9 ± 3.9 |
|  | Bing | 89 ± 9.5 | 48.2 ± 2.7 |
|  | ChatGPT | 244 ± 57.8 | 41.4 ± 4.7 |
| What is an electrical storm? | Bard | 311 ± 32.5 | 63.4 ± 4.4 |
|  | Bing | 153 ± 0.0 | 47.8 ± 0.3 |
|  | ChatGPT | 289 ± 48.2 | 45.1 ± 1.5 |
| Living with an CIED |  |  |  |
| Can I perform an MRI with an implanted pacemaker? | Bard | 363 ± 29.1 | 55.3 ± 4 |
|  | Bing | 168 ± 2.5 | 33.6 ± 8 |
|  | ChatGPT | 315 ± 37.6 | 26.1 ± 5.7 |
| I have an implanted cardioverter-defibrillator. Can I have an active sexual life? | Bard | 90 ± 125.9 | 66 ± 15 |
|  | Bing | 118; 29.0 | 52.3 ± 4.8 |
|  | ChatGPT | 370 ± 11.8 | 34.9 ± 4 |
| I have an implanted cardioverter-defibrillator. Do I have any limitations on driving? | Bard | 333 ± 37.0 | 64.9 ± 5.1 |
|  | Bing | 205 ± 31.1 | 41.6 ± 0.6 |
|  | ChatGPT | 328 ± 20.6 | 31.6 ± 6.3 |
| I have an implanted cardioverter-defibrillator. Can I work out? | Bard | 341 ± 12.5 | 54.6 ± 3.4 |
|  | Bing | 162 ± 33.5 | 31.2 ± 6.9 |
|  | ChatGPT | 405 ± 20.3 | 34.2 ± 2.2 |
| I have been implanted with a cardioverter-defibrillator. Can I use an induction stove? | Bard | 250 ± 75.3 | 55.3 ± 3.2 |
|  | Bing | 100 ± 8.5 | 51.5 ± 1.3 |
|  | ChatGPT | 329 ± 4.9 | 30.8 ± 3.6 |
| I have an implanted cardioverter-defibrillator. Can I use my cellphone? | Bard | 223 ± 32.0 | 60 ± 6 |
|  | Bing | 127 ± 25.8 | 44.9 ± 11.8 |
|  | ChatGPT | 302 ± 36.0 | 39.1 ± 6.2 |
| Can I bath or swim with an implantable cardioverter-defibrillator? | Bard | 195 ± 5.9 | 66.2 ± 3.8 |
|  | Bing | 63 ± 6.1 | 35.4 ± 4.1 |
|  | ChatGPT | 328 ± 33.7 | 40 ± 7.3 |
| I have an implanted pacemaker. Can I undergo radiation therapy for my prostate cancer? | Bard | 13 ± 0.6 | 78.3 ± 3.1 |
|  | Bing | 189 ± 25.9 | 39.4 ± 2.5 |
|  | ChatGPT | 280 ± 39 | 8.1 ± 4.2 |
| Can I die with a pacemaker? | Bard | 177 ± 137.5 | 51.6 ± 8.2 |
|  | Bing | 205 ± 62.0 | 48.5 ± 4.1 |
|  | ChatGPT | 309 ± 21.0 | 29.8 ± 2.6 |
| Can I use an electric car with my implantable cardioverter-defibrillator? | Bard | 273 ± 41.9 | 51.9 ± 4.3 |
|  | Bing | 107 ± 1.0 | 26.7 ± 2.1 |
|  | ChatGPT | 329 ± 4.6 | 26.7 ± 4.2 |

ICD – implantable cardioverter defibrillator, CRT – cardiac resynchronization therapy, CRT-D – cardiac resynchronization therapy with defibrillator, CRT-P – cardiac resynchronization therapy with pacemaker

Supplementary Table S5: Examples of confabulation and inappropriate context given by natural language processing chatbots.

| **Question** | **Response** |
| --- | --- |
| Can I bath or swim with an ICD? | If you are going to be swimming in the ocean, rinse your ICD off with fresh water after swimming to remove any salt or sand residue. Avoid using jets or sprayers on your ICD. Pat your ICD dry with a towel after bathing or swimming.  Avoid swimming in salt water, as the salt can corrode the leads of your ICD. |
| I have an ICD. Do I have any limitations on driving? | Here are some additional tips for driving safely with an ICD: Keep your ICD at all times. |
| My pacemaker pocket is red and swollen. What shall I do? | Apply warm compresses to the area for 20 minutes at a time, several times a day.  Elevate the area when possible. |
| What is remote monitoring for CIEDs? | Remote monitoring for CIEDs typically works through a small device that the patient wears at home. |
| When do I need transesophageal echocardiography before an electrical cardioversion? | On the day of your TEE, you will be given a sedative to help you relax. A thin tube with a camera on the end will be inserted into your throat and down into your esophagus. The camera will be used to take pictures of your heart. |

TEE – transesophageal echocardiography, CIED – cardiac implantable electronic devices, ICD – implantable cardioverter defibrillator.
